# Modulation of acoustic waves by a broadband metagrating

**DOI:** 10.1038/s41598-019-43850-y

**Published:** 2019-05-13

**Authors:** Yihe Wang, Ying Cheng, Xiaojun Liu

**Affiliations:** 1 Key Laboratory of Modern Acoustics, Department of Physics and Collaborative Innovation Center of Advanced Microstructures, Nanjing University, 210093 Nanjing, China; 2 State Key Laboratory of Acoustics, Institute of Acoustics, Chinese Academy of Sciences, 100190 Beijing, China

**Keywords:** Metamaterials, Acoustics

## Abstract

Metasurface has recently attracted a lot of attentions for controlling wave fields. Based on the diffraction effects of phase gratings, we demonstrate a broadband acoustic metagrating which can concentrate the diffracted waves in the first (±1) orders and achieve multifunctional wave steering such as broadband anomalous diffraction. In the acoustic metagrating, the subwavelength rectangular waveguides (SRWs) function as the periodic elements to replace the fences in ordinary gratings. Thus, we can achieve a group of phase delay from 0 to 2π independently with frequency just by reconfiguring the relative locations of the effective apertures. With the iterative algorithm, the acoustic metagrating can be used to record the phase profile and then control the output waveform. We further demonstrate that the broadband metagrating can be used to achieve the acoustic Gaussian beam. By rotating the periodic elements into a two-dimensional structure, the Bessel beam is further obtained.

## Introduction

Acoustic metasurface is composed of periodic subwavelength elements which can exhibit untraditional manipulation of local and far-field sound pressure distributions. When acoustic waves reach and react with metasurface, phase and amplitude should be modulated, and then the trace of incident waves can be manipulated artificially. By properly designing the positions of periodic elements, metasurface can achieve multifunctional steering of acoustic waves, i.e., acoustic focusing^1–6^, acoustic carpet cloaking^7–9^, asymmetric acoustic transmission^10,11^, acoustic trapping^12,13^, acoustic holography^13–15^, sound vortices^16^ and Mie resonance^17^. However, most of the reported metasurface have certain limiting factors, i.e., the narrow work frequency band. For example, in order to control wave trace, the metasurface must provide corresponding phase profile in different positions. This phase profile is usually derived from the generalized laws of diffraction^18^, leading to the inherent dependence on the working frequency. Thus, the phase aberrations make the metasurface only work in a narrow frequency band. Recently, as an emerging kind of metasurface, the artificial metagratings have received much attentions due to their more combination functions and more advantage performance over ordinary phase gratings, such as the enhanced acoustic transmission through a rigid plate^19^, directional beam forming^20^, and asymmetric acoustic transmission^21^. In fact, a new broadband optical metagrating has been proposed to achieve anomalous wave steering by engineering diffraction optical gratings^22^. Based on the grating equation, the frequency-dependent specialty can be easily eliminated in the metagratings.

In this paper, we demonstrate a broadband acoustic metagrating by reconfiguring the typical diffraction effects. The metagrating is composed of periodic structured elements consisted by subwavelength rectangular waveguides (SRWs) to achieve the steering of acoustic waves. It is found that the metagrating can convert the normally incident waves into two symmetrical directions in a wide frequency band and provide a phase delay independently with frequency. We further determine the multifunction of the metagrating in wave steering, such as achievement of acoustic Gaussian and Bessel beams.

## Results

### The diffraction intensity characteristics of grating

**Figure 1:**
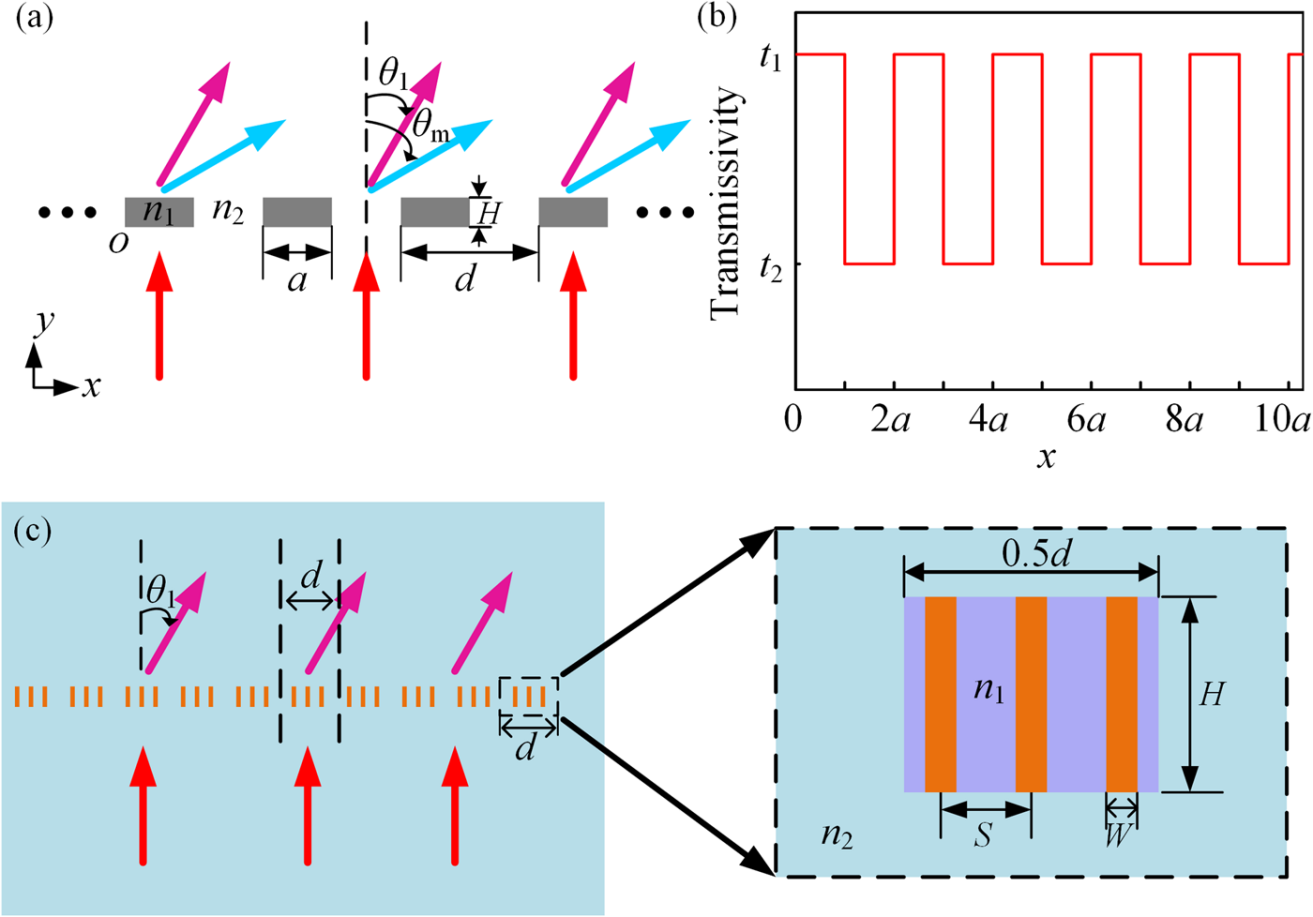
(**a**) Schematic diagram of a regular phase grating. The grating constant *d* is fixed as twice of the aperture size a. *H* is the height of the fence. *θ*_1_ and *θ*_m_ represent the angle of the 1st order and the *m*th order diffraction, respectively. (**b**) Transmissivity at different positions of the grating. Point *O* in (**a**) denotes the origin of the coordinate and we take five periods in the positive direction of *x*-axis. *t*_1_ and *t*_2_ qualitatively represent the transmissivity of the fence and aperture, respectively. They are related to the material parameters of fence and aperture. (**c**) Schematic diagram of the acoustic metagrating. Inset: zoom-in view of a basic element. This metagrating can replace the regular phase grating and concentrate the transmitted waves propagation in the first orders. The effective area of *n*_1_ is shown in purple. The width of the purple region is 0.5*d* and the height is the same as the height of waveguide *H*.

First, we start from the transmitted intensity through the phase grating. Figure 1(a) shows the schematic diagram of a regular phase grating. When plane waves are normally incident onto the grating, the angles of diffracted waves satisfy the grating equation of $\sin \,{\theta }_{m}=m\lambda /d$, where *m* $(\,=\,0,\,\pm \,1,\pm \,2,\,\ldots )$ represents the diffraction order, *θ*_m_ is the diffraction angle of the *m*th order diffraction, *λ* is the wavelength of incident waves and *d* is the grating constant. Here, the grating constant *d* is fixed as twice of the aperture size *a*, i.e., *d* = 2*a*. Based on the aperture angular spectrum theory^23^, when plane waves of unit amplitude are normally incident to the phase grating, the angular spectrum of diffraction pressure just crossing the grating is equal to the Fourier transform of the grating’s transmissivity function *t*, and then the angular spectrum of diffraction intensity can be the square of Fourier transform *F*(*t*). The grating’s transmissivity *t*(*x*) can be obtained as a combination of that for the fences and apertures. When plane waves cross the phase grating, the fences and apertures should result in different phase delay $\,{\phi }_{1}=2\pi {n}_{1}H/{\rm{\lambda }}$ and $\,{\phi }_{2}=2\pi {n}_{2}H/{\rm{\lambda }}$, respectively. Thus, as shown in Fig. 1(b), the transmissivity function *t*(*x*) of the grating can be expressed as

1 $$t(x)=\{\begin{array}{cc}{e}^{i2\pi {n}_{1}H/\lambda }, & nd < x < nd+a\\ {e}^{i2\pi {n}_{2}H/\lambda }, & nd+a < x < (n+1)d\end{array}\,(n=0,\,1,\,2,\,\ldots )$$

where *n*_1_ and *n*_2_ are the diffraction indexes of the fence and aperture, respectively, and *H* is the height of the fence. Based on Fourier expansion for *t*(*x*), we can get

2 $$t(x)=\sum _{m=-\infty }^{\infty }{c}_{m}{e}^{\frac{im\pi x}{a}}.$$

Here,$\,{c}_{m}=\frac{1}{2a}{\int }_{0}^{2a}t(x){e}^{-im\pi x/a}dx$. Then the angular spectrum of the *m*th diffraction intensity is

$$\begin{array}{c}\begin{array}{rcl}I({f}_{x})={|F[t(x)]|}^{2} & = & {|F[\sum _{m=-\infty }^{\infty }{c}_{m}{e}^{\frac{im\pi x}{a}}]|}^{2}={|\sum _{m=-\infty }^{\infty }{c}_{m}F[{e}^{imKx}]|}^{2}\\  & = & {|\sum _{m=-\infty }^{\infty }{c}_{m}F[{e}^{i2\pi \frac{mK}{2\pi }x}]|}^{2}={|\sum _{m=-\infty }^{\infty }{c}_{m}\delta ({f}_{x}-\frac{mK}{2\pi })|}^{2}\\  & = & {|\sum _{m=-\infty }^{\infty }{c}_{m}\delta ({f}_{x}-\frac{m}{2a})|}^{2}\end{array}\end{array}$$

Here,$\,{f}_{x}=\frac{\sin \,{\theta }_{m}}{\lambda }$ and $K=\frac{2\pi }{2a}=\frac{\pi }{a}$ represents the magnitude of the grating vector. Then it can be obtained by combining the grating equation

3 $$I({f}_{x})={|{c}_{m}|}^{2}=\{\begin{array}{ll}{|\frac{1}{2a}{\int }_{0}^{a}{e}^{i\frac{2\pi {n}_{1}H}{\lambda }}dx+\frac{1}{2a}{\int }_{a}^{2a}{e}^{i\frac{2\pi {n}_{2}H}{\lambda }}dx|}^{2} & ,\,m=0\\ {|\frac{1}{2a}{\int }_{0}^{a}{e}^{i\frac{2\pi {n}_{1}H}{\lambda }}{e}^{\frac{-im\pi x}{a}}dx+\frac{1}{2a}{\int }_{a}^{2a}{e}^{i\frac{2\pi {n}_{2}H}{\lambda }}{e}^{\frac{-im\pi x}{a}}dx|}^{2} & ,\,m\ne 0\end{array}\,=\{\begin{array}{ll}{\cos }^{2}[\frac{\pi H}{\lambda }({n}_{1}-{n}_{2})], & m=0\\ \frac{{[{(-1)}^{m}-1]}^{2}}{{m}^{2}{\pi }^{2}}{\sin }^{2}[\frac{\pi H}{\lambda }({n}_{1}-{n}_{2})], & m\ne 0\end{array}$$

From Eq. (3), the intensities of 0th and 1st orders are $\,{I}_{0}={\cos }^{2}\,[\frac{\pi H}{\lambda }({n}_{1}-\,{n}_{2})]$ and ${I}_{1}=\frac{4}{{\pi }^{2}}\,\sin \,{}^{2}\,[\frac{\pi H}{\lambda }({n}_{1}-{n}_{2})]$, respectively. Note that the intensities of the other order diffractions (*m* = 2, 3….) can be also determined by Eq. (3). According to the diffraction characteristics of an ordinary grating^24^, the intensity of *m*th order diffraction decreases with the increases of diffraction order *m*. From Eq. 3, we know that the intensity on the even diffraction order is 0 and the intensity on 3rd diffraction order is ${I}_{3}=\frac{4}{9{\pi }^{2}}\,{\sin }^{2}\,[\frac{\pi H}{\lambda }({n}_{1}-{n}_{2})]=\frac{1}{9}{I}_{1}$. It is obvious that the intensity on 3rd diffraction order is small and negligible. The intensity on other orders is less than 3rd diffraction order, then the intensity on the order ${\rm{m}} > 1$ can be neglected. Obviously, the energies of the zero and first order diffractions take up majority energy of the diffracted waves. When the grating’s parameters meet certain conditions resulting in *I*_0_ = 0, i.e.,

4 $${n}_{1}-{n}_{2}=(k+\frac{1}{2})\lambda /H,\,(k=0,\,\pm \,1,\,\pm \,2,\,\ldots )$$

the 0th order diffraction is totally suppressed and the diffracted waves should mainly distribute in the 1st orders.

### Design of the acoustic metagrating

As shown in Fig. 1(c), the metagrating can be constructed by implementing artificial reconfiguration of diffraction intensity based on above functions. Here, each fence of the grating shown in Fig. 1(a) is replaced by three subwavelength rectangular waveguides (SRWs). When the effective diffraction index of the metagratings *n*_1_ (the effective area of *n*_1_ is shown in purple in Fig. 1(c). The width of the purple region is 0.5*d* and the height is the same as the height of waveguide *H*) and the ambient medium *n*_2_ satisfy Eq. (4), most of the waves crossing the metagrating should propagate along the direction $\,{\theta }_{1}={\sin }^{-1}(\frac{\lambda }{d})$. It is obvious that plane waves with different wavelengths diffract in different directions. To be specific, trapezoidal, triangular, rectangular or other shapes of waveguides can be used to replace the fences of grating as long as we choose proper parameters of waveguides to make the effective diffraction index of metagrating and the ambient medium satisfy Eq. (4). In Fig. 1(c), the distance, width and height of a waveguide are set as *S* = 0.16*λ* ~ 0.22*λ*, *W* = 0.05*λ* ~ 0.07*λ*, *H* = 0.33*λ* ~ 0.43*λ*, respectively. By choosing materials with the proper parameters, the metagrating can play a role like beam splitter and the angle of diffracted waves is proportional to the wavelength according to the grating equation.

### Diffraction characteristics of the metagrating

**Figure 2:**
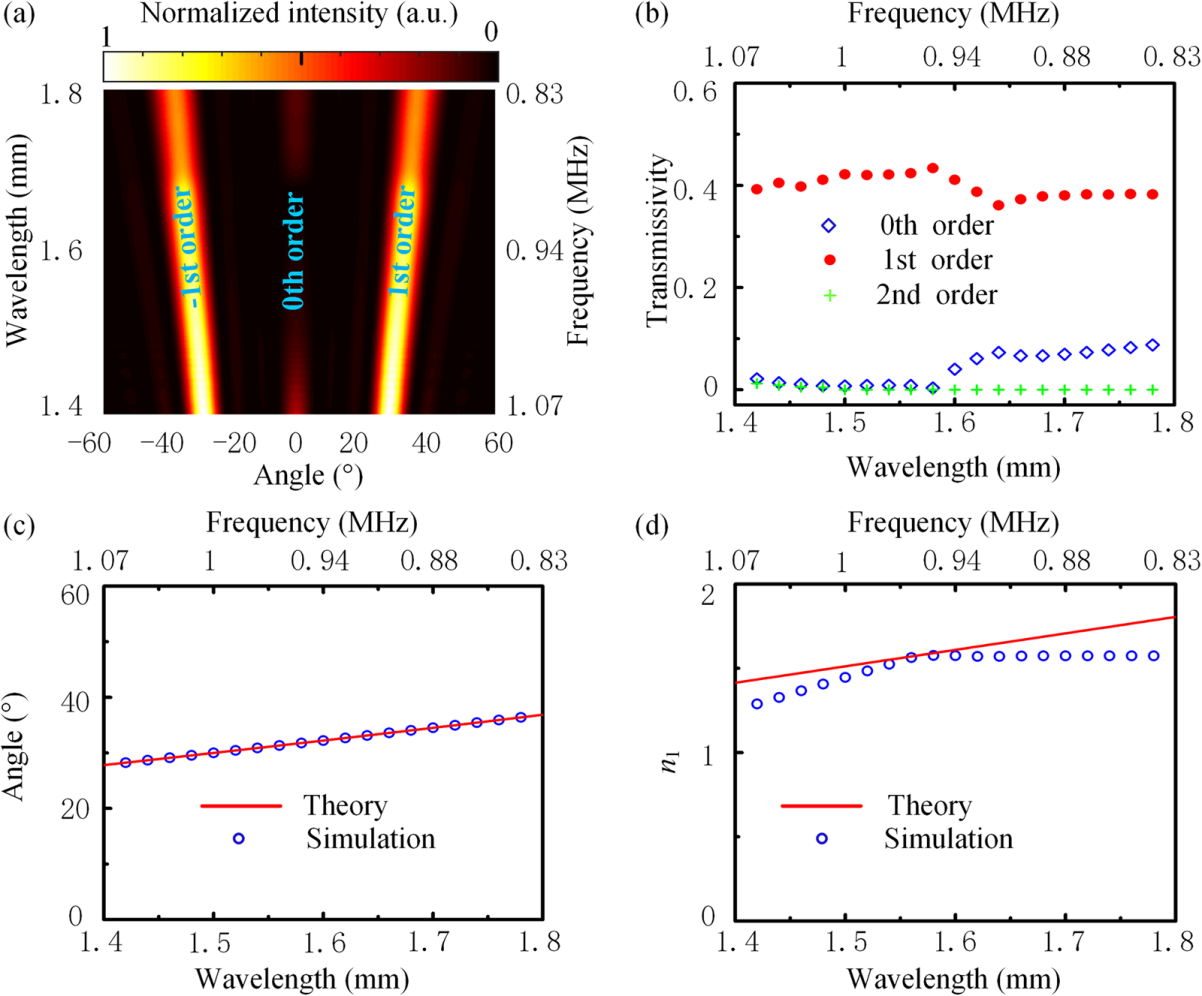
(**a**) Far-field intensity distribution of diffracted waves at different incident wavelengths. (**b**) Transmissivity of 0th-order, 1st-order, 2nd-order diffracted waves, respectively. The diffracted waves propagate in two symmetrical directions, and the maximum transmissivity of each 1st order diffraction reaches 0.5. (**c**) The 1st order diffraction angle in theory and simulation. (**d**) The effective diffraction index of metagrating (blue hollow circle) and the required diffractive index (red line).

The far-field intensity distribution exhibits that the diffracted waves mainly distribute in two symmetrical directions, and almost no wave is found in the directions of other orders, as shown in Fig. 2(a). In the simulations, the parameters of SRW are set as *S* = 0.3 mm, *W* = 0.1 mm, *H* = 0.6 mm, and *d* = 3 mm, respectively, and the incident wavelengths are in the range of 1.4 ~ 1.8 mm. Figure 2(b) shows the transmissivity of different orders. The transmissivity of negative orders is equal to the corresponding positive orders because of symmetry. It is found that the diffracted waves in the first orders (+1, −1) take up more than 80% of the total diffraction energy. As shown in Fig. 2(c), the direction of transmission waves is consistent with that of the 1st order diffraction. We further use the effective diffraction index theory^25^ to calculate the effective diffraction index of the metagrating (*n*_1_). Under the same incident wavelength, as shown in Fig. 2(d), it is found that achieved *n*_1_ agrees well with the theoretical value derived from Eq. (4) at *k* = 0. When *n*_1_ and *n*_2_ satisfy Eq. (4), the phase difference between the 0th order waves crossing the SRWs and ambient medium is $\frac{2\pi H}{\lambda }({n}_{1}-{n}_{2})=\pi$. Consequently, the 0th order diffraction is suppressed while acoustic waves are mostly distributed in the 1st orders. Simpler structures, i.e., one or two SRWs can also be used to achieve the 0th-order suppressing effect, but have the following disadvantages. If one SRW is used to replace the fence, the width of waveguide should be 1.5 mm according to the condition of Eq. (3), which is as long as one wavelength. In order to obtain the same phenomenon with the same frequency band as above, the parameterized scanning calculated by COMSOL shows that *H* should be 0.35 mm. Then the area of SRW is about three times as much as when using three SRWs. Moreover, when using two SRWs, the frequency band is found to be narrowed by scanning three parameters (*W*, *S*, *H*). For example, when *W* = 0.8 mm, *S* = 0.3 mm, *H* = 0.43 mm, the 0th-order suppressing effect was limited to the wavelength range of 1.5 mm ~1.6 mm. To sum up, the metagrating can concentrate the transmitted waves in the 1st orders under a wide work frequency range. Thus, by setting proper parameters of the SRW (e.g., *W*, *H*, *S*), the metagrating can achieve broadband anomalous diffraction^26^, and then can be used to work as a beam splitter.

### Implementation of Gaussian beam

**Figure 3:**
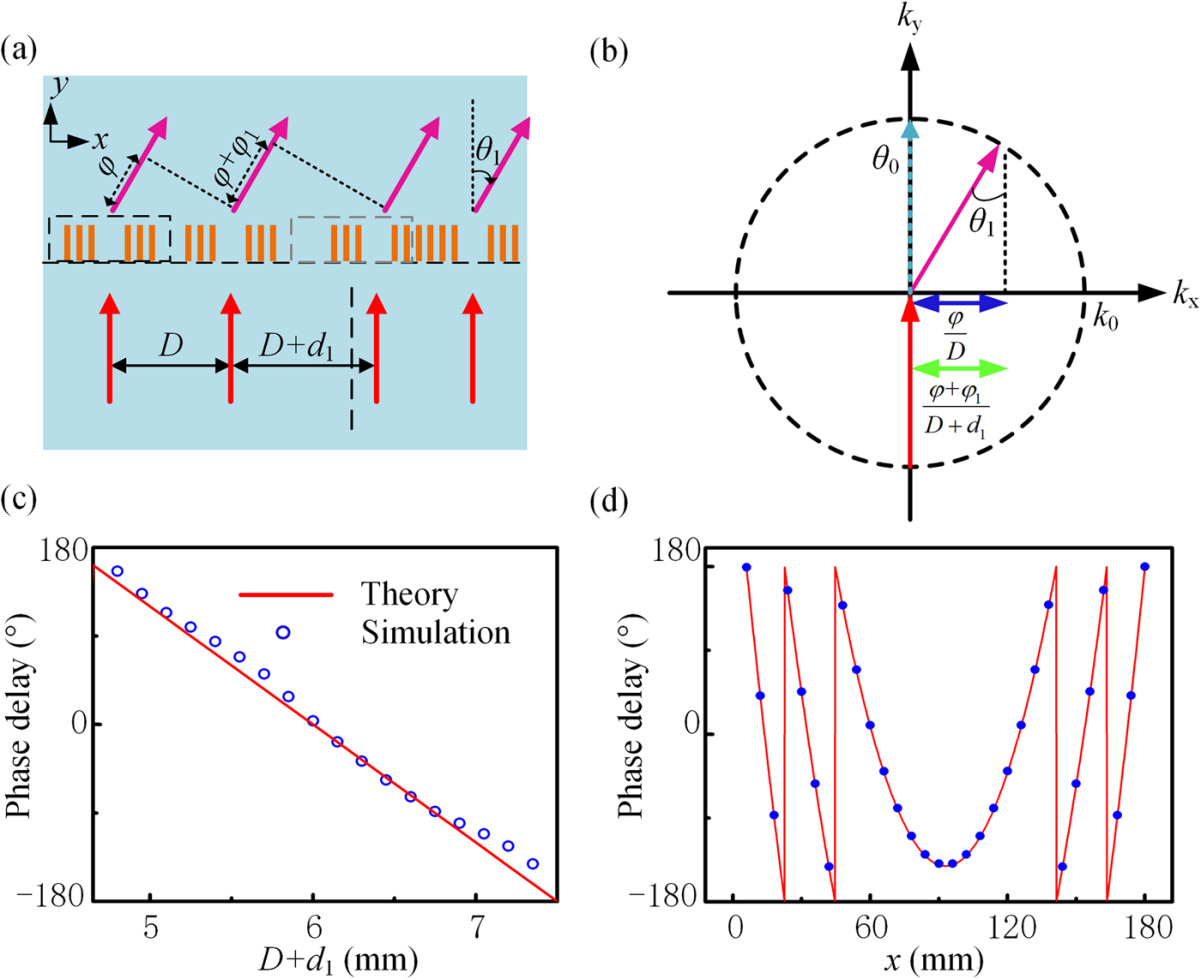
(**a**) Schematic diagram of effective apertures. Each aperture of the metagrating is composed of six SRWs. *φ* and *φ*_1_ represent the phase delay caused by the path difference *D*sin*θ*_1_ and *d*_1_sin*θ*_1_, respectively. (**b**) The momentum relation between the 0th and 1st-order diffracted waves. *θ*_0_ and *θ*_1_ represent the diffracting angle of 0th and 1st orders, respectively. It is found that the transverse momentum imparted by the displacement of apertures remains the same. The radius of the dashed circle is *k*_**0**_ = 2π**/***λ*. (**c**) Phase delay varies from 0 to 2π when the distance between effective apertures changes. (**d**) Phase distribution of metagrating to implement the Gaussian beam. The red line represents the theoretical phase and the blue circle represents the discretized phases of thirty pairs of SRWs.

Expect for anomalous diffraction, the metagrating can also be used to achieve the acoustic Gaussian beam. As shown in Fig. 3(a), six SRWs function as the effective aperture. In the metagrating, the displacement of effective aperture leads to the variation of acoustic path difference and thus causes the phase delay *φ*_1_. In the momentum space, as shown in Fig. 3(b), different displacements of effective apertures result in the same transverse momentum, i.e.,$\,\frac{\phi +{\phi }_{1}}{D+{d}_{1}}=\frac{\phi }{D}$. Here *D* represents twice the grating constant, i.e., *D* = 2*d*, $\phi =4\pi$ represents the phase delay caused by the path difference *D*sin*θ*_1_, *d*_1_ represents the displacement of effective aperture. Then we can get the relation of phase delay and displacement *d*_1_, i.e.,

5 $${\phi }_{1}=\phi \frac{{d}_{1}}{D}$$

It is noted a group of phase delay *φ*_1_ ranging from 0 to 2π can be obtained by altering *d*_1_, and *φ*_1_ is independent with frequency. The simulated results in Fig. 3(c) show that the phase delay covers from 0 to 2π and varies linearly with the distance variation *d*_1_. Thus, the metagrating can record the phase information of designed waveform. The required phase profile of metagrating is obtained by the Gerchberg-Saxton phase-retrieval with iterative angular spectrum algorithm^27^. In this algorithm when the waveform is known, the corresponding phase distribution in the metagrating can be obtained through several iterations. With the phase distribution, the displacement *d*_1_ in different positions is also available from Eq. (5). By arranging the effective apertures according to *d*_1_, the metagrating can be constructed. When the plane waves are normally incident onto the metagrating, the wanted waveform pattern should be obtained in the output side.

**Figure 4:**
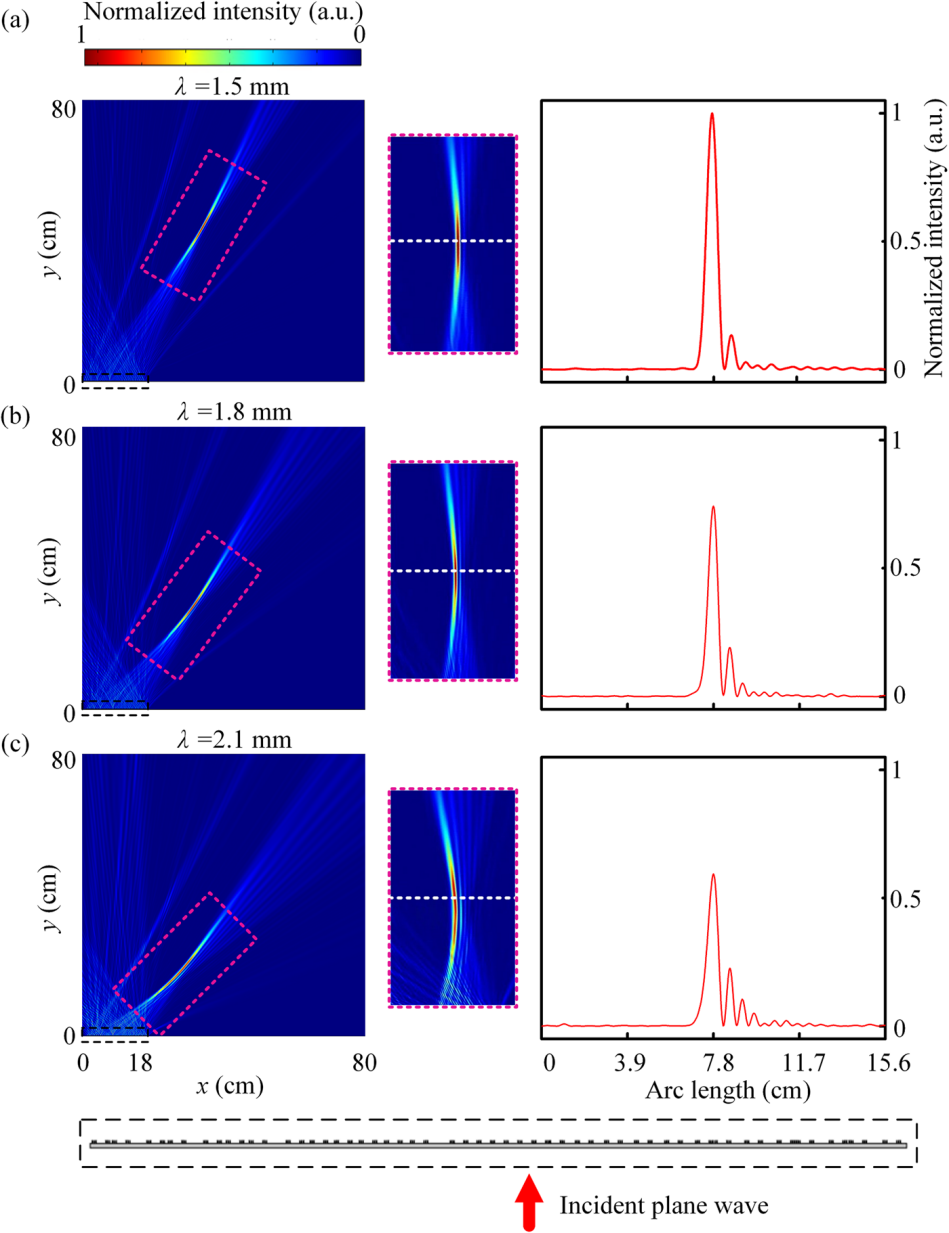
Intensity distribution of the Gaussian beam when the incident wavelengths are (**a**) 1.5 mm, (**b**) 1.8 mm, (**c**) 2.1 mm, respectively. The middle panels are the zoom-in views of the purple dashed box in the left panels and the white dashed lines indicate the position of the beam waist. The right panels show the intensity distribution in the beam waist position. The black dashed box indicates the metagrating and its enlarged view.

Based on the above method of phase control, the plane waves can be converted to the Gaussian beam. Figure 4 shows the simulated results when the incident wavelengths are 1.5 mm, 1.8 mm, 2.1 mm, respectively. In the metagrating, thirty pairs of SRWs are used to implement the Gaussian beam. The arrangement of periodic elements should be obtained by the above iterative algorithm. The Gaussian beam can be viewed as get a one-dimensional image of the gaussian at the position of its beam waist. Here, we set the width of the waist position of the Gaussian beam at the image plane to be about four times the wavelength at *λ* = 1.5 mm. The image plane is perpendicular to the direction of the 1st order diffraction and its length is equal to the projection of the entire metagrating in the diffraction direction. Figure 3(d) shows the theoretical phase distribution of metagrating to implement the Gaussian beam. Then the displacement *d*_1_ of each element in different positions is also available from Eq. (5). By arranging the effective apertures according to *d*_1_, the metagrating can be constructed and its details is shown in the black dashed box in Fig. 4. In arrangement, if two adjacent effective apertures overlap, only the waveguide in the second effective aperture will be retained. Then large errors will be caused in achieving the Gaussian beam if one or two SRWs are used to replace each fence of the grating. The right panels in Fig. 4(a–c) show the intensity distribution at the beam waist position and the waist position is indicated by a white dashed line. The length of these white dashed lines are the same, and the length is fixed at the width of image plane when the incident wavelength is 1.5 mm. It is found that the Gaussian beam is obtained in different directions when the incident wavelength changes, but the beam remains approximately the same width at the waist position. Thus, the metagrating is broadband to achieve one dimensional images.

### Implementation of Bessel beam

Finally, we rotate the elements to get a two-dimensional structure, which can create the “non-diffracting” Bessel beam^28^ and the Bessel beam can be used to achieve microparticle manipulations^29^. An ideal 0th order Bessel beam can be described by

6 $$P(r,y)={P}_{0}{J}_{0}({k}_{r}r){e}^{i{k}_{y}y}$$

Where *P*(*r*, *y*) is amplitude in the radial distance *r* and the axial distance *y*, *J*_0_(*k*_*r*_*r*) is the zero order Bessel function, and ${k}_{r}({k}_{r}=\sqrt{{k}_{x}^{2}+{k}_{z}^{2}}),\,{k}_{y}\,$,are radial and axial wave vectors, respectively. Then we can get the intensity at the position (*r*, *y*)

7 $$I(r,y)=|{P}_{0}{J}_{0}({k}_{r}r){|}^{2}$$

**Figure 5:**
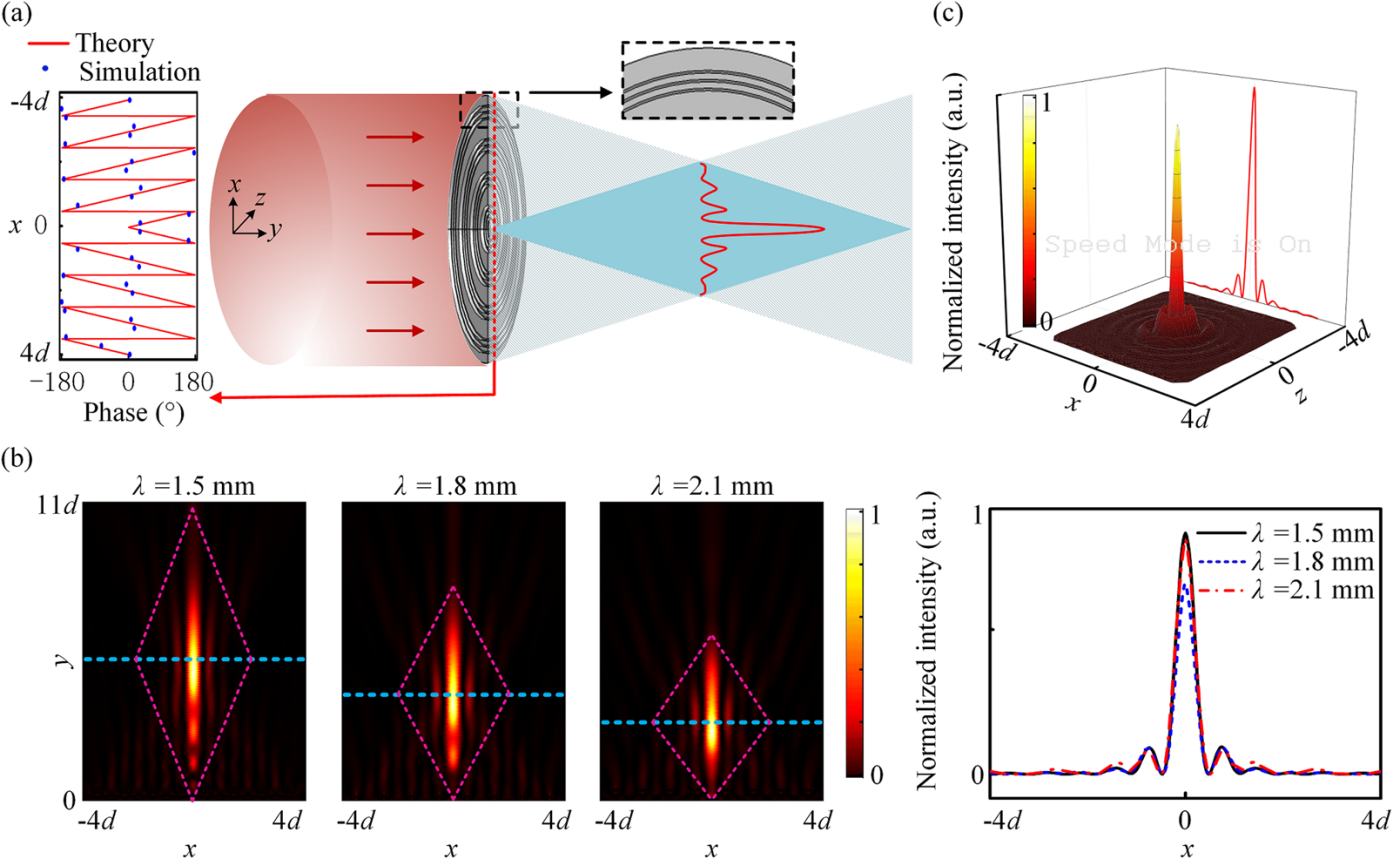
(**a**) Schematic diagram to form the Bessel beam. When a set of plane waves are diffracted by the metagrating in *θ* and − *θ* angle symmetrically along *y-*axis, the transmitted waves will form the Bessel beam in the output side. The black dashed box shows the zoom-in details of the metagrating. Left panel: the theoretical phase distribution (red line) and discretized phases of disc metagrating (blue circle). (**b**) The Bessel beam formed when the incident wavelengths are 1.5 mm, 1.8 mm, 2.1 mm, respectively. (**c**) The top panel shows the intensity distribution in the cross section at *y* = 5.5*d* when the incident wavelength *λ* = 1.5 mm. The bottom panel shows the intensity distribution along the blue dashed line in (**b**), of which the corresponding positions are *y* = 5.5*d*, 4*d*, 3*d* for *λ* = 1.5, 1.8, and 2.1 mm, respectively.

For Bessel function, the cross-section intensity *I* (*r*, *y*) is changeless along the axial direction (*y*-direction) and composed of a set of concentric annulus. Indeed, the Bessel beam can be seen as a cluster of plane waves going through a cone with taper angle $\,\theta ={\tan }^{-1}(\frac{{k}_{r}}{{k}_{y}})$. Thus the beam is just like an interference field composed of a cluster of iso-amplitude plane waves with the different azimuth angles but identical intersection angle with the axial direction. Then we can use the above-mentioned metagrating to get Bessel beam by rotating the waveguides to obtain a disc metagrating composed of a set of toroidal waveguides. The metagrating can divide the normally incident waves into two symmetrical directions centered on the *y* axis. The symmetrical transmitted waves will intervene in the intersection region and then form the Bessel beam, as shown in Fig. 5(a). The inset of Fig. 5(a) shows the phase distribution derived from the generalized laws diffraction of theoretical metagrating (red line) and the discretized phases of disc metagrating (blue circle). Note that the metagratings can provide the phase distribution to implement the Bessel beam. Figure 5(b) shows the intensity profile of the vertical section at *z* = 0 when the incident wavelengths are 1.5 mm, 1.8 mm, 2.1 mm, respectively. It is found that the smaller the wavelength is, the farther it propagates. The area in rhombus exhibits the waves which concentrate in a small area and propagate a long distance with shape changeless. The radius of the plane waves is about 8*λ* while the diffracted waves can propagate non-diffractively over 20*λ* long when the wavelength is 1.5 mm. The top panel of Fig. 5(c) takes the incident wavelength of 1.5 mm as an example, which shows the intensity distribution at the cross section at *y* = 5.5*d*. The diffracted waves in the cross section exhibit water-ripples-like shape, which are composed of a center peak and a set of side-lobes in concentric circles. The envelope line of the projection is just like the 0th order Bessel function curve in Fig. 5(a). The bottom panel in Fig. 5(c) shows the intensity distribution of the blue dashed line in Fig. 5(b) at *y* = 5.5*d*, 4*d*, 3*d* when the incident wavelengths are 1.5 mm, 1.8 mm, 2.1 mm, respectively. Although the intensity at the center position varies with the incident wavelength, the contour of the curves always conform to the Bessel curve, which confirm that the disc metagrating can convert the incident waves to the Bessel beam in a wide frequency work band.

## Discussion

We have proposed a broadband acoustic metagrating. By reconfiguring the diffraction effects through the distributed SRWs, the acoustic metagrating can achieve broadband multifunctional wave steering such as anomalous diffraction in a wide frequency band. Besides, by arranging the SRWS according to the diffraction effects of irregular gratings, the metagratings can also provide a group of phase delay from 0 to 2π, which is only related to the grating constant and the displacement of effective apertures. Thus, the metagratings can modulate the wave trace in a wide frequency band, and convert the plane waves to the Gaussian beams or Bessel beams at different incident wavelengths. The proposed metagrating may provide a possibility to the broadband acoustic devices.

## Methods

To demonstrate the versatility of the metagrating, the full wave simulations are performed with the COMSOL Multiphysics based on the finite element analysis (FEA) method. In the simulation, water (*c*_w_ = 1,500 m/s, *ρ*_*w*_ = 1 × 10^3^ kg/m^3^, *n* = 0.23) and rubber (*c*_*rL*_ = 336 m/s, *ρ*_*r*_ = 0.89 × 10^3^ kg/m^3^) are used as the ambient medium and the material of subwavelength rectangular waveguides, respectively. When calculating the far-field intensity distribution in Fig. 2(a), the SRWs with one period is selected and the periodic boundary conditions are applied on left and right sides. In order to get the transmission coefficient of different orders in Fig. 2(b), twenty periods of SRWs are used in simulation. By combining the grating equation and integrating the intensity around the structure, the total intensity and the intensity of different diffraction angles can be obtained. Then the transmission coefficient can be calculated.

## Data Availability

The datasets generated during and/or analyzed during the current study are available from the corresponding author on reasonable request.
